# Late presentation of traumatic tricuspid valve chordal rupture and pericardial rupture with cardiac herniation: a case report

**DOI:** 10.1186/s12872-024-03716-2

**Published:** 2024-01-13

**Authors:** Nebojsa Radovanovic, Maja Prodanovic, Mina Radosavljevic-Radovanovic, Ilija Bilbija, Olga Petrovic, Nina Lojovic, Emilija Kecman, Aleksandar Djekic, Milos Radovanovic, Dragan Matic

**Affiliations:** 1 Cardiology Clinic, University Clinical Center of Serbia, Dr Koste Todorovića 8, Belgrade, 11000 Serbia; 2 Cardiac Surgery Clinic, University Clinical Center of Serbia, Belgrade, Serbia; 3 Faculty of Medicine, University of Belgrade, Belgrade, Serbia

**Keywords:** Tricuspid valve, Chordal rupture, Pericardial rupture, Cardiac herniation, Trauma

## Abstract

**Background:**

Although chest trauma happens very often, accompanying tricuspid valve injuries occur rarely and may be manifested by scarce symptoms and signs. Pericardial rupture with cardiac herniation is even a bigger rarity. Transthoracic echocardiography plays a key role in the diagnosis of valve injuries but is of limited value in cardiac herniation.

**Case presentation:**

We present the case of 58-year-old man who experienced severe chest trauma in a car accident. Symptoms of right heart failure occurred 10 years after the injury, due to the loss of tricuspid leaflet support caused by the rupture of tendinous chords with significant tricuspid regurgitation. Intraoperatively, old posttraumatic pericardial rupture into left pleura was also found, with partial cardiac herniation and pressure of the edge of pericardium on all left-sided coronary arteries simultaneously. The patient was successfully operated and is free of symptoms 4 years later.

**Conclusions:**

This case emphasizes the importance of timely diagnosis and underlines a mechanism that leads to delayed rupture of the tricuspid valve apparatus. Repeated echocardiography in all patients who experienced chest trauma could be of great importance. Also, given the limited value of echocardiography in posttraumatic pericardial rupture and cardiac herniation, cardiac computed tomography should be performed.

**Supplementary Information:**

The online version contains supplementary material available at 10.1186/s12872-024-03716-2.

## Background

After a chest trauma, patients often have a wide range of chest injuries. Therefore, injuries of the myocardium and valvular apparatus, if not clinically manifested immediately, can remain unrecognized for a long period of time. Traumatic tricuspid regurgitation is usually well tolerated in the acute phase, which is why surgical treatment of the tricuspid valve is performed much later than the onset of the injury. Transthoracic echocardiography (TTE) plays an important role in the diagnosis of valvular apparatus injuries, thus enabling early adequate treatment. A very small number of pericardial ruptures caused by chest wall trauma has been described in clinical practice, with various presentations. We present the case of a patient who was injured in a traffic accident, and manifested signs and symptoms of tricuspid valve and pericardial rupture with partial cardiac herniation 10 years later.

## Case presentation

**Fig. 1:**
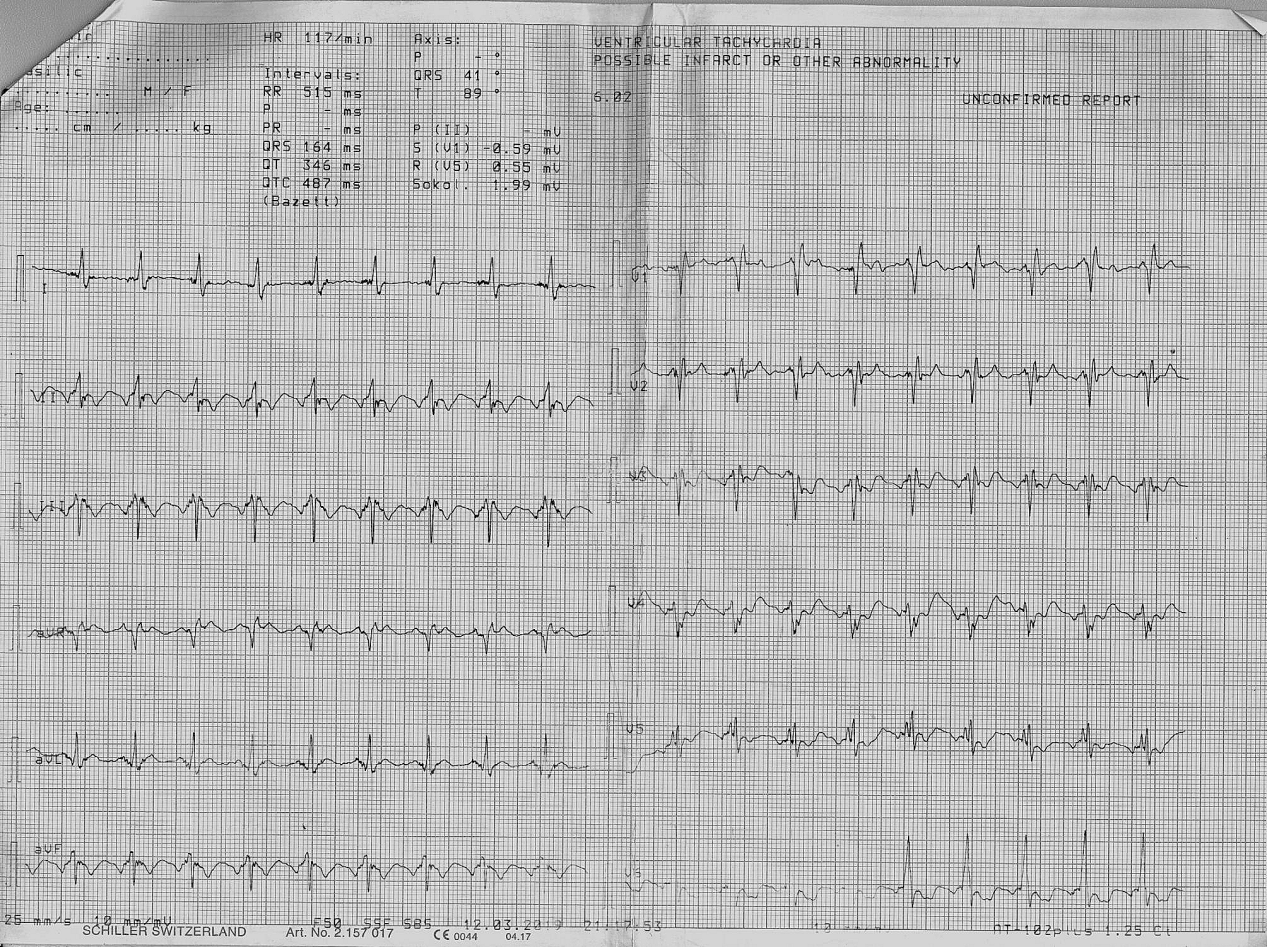
ECG on admission showing right bundle branch block

**Fig. 2:**
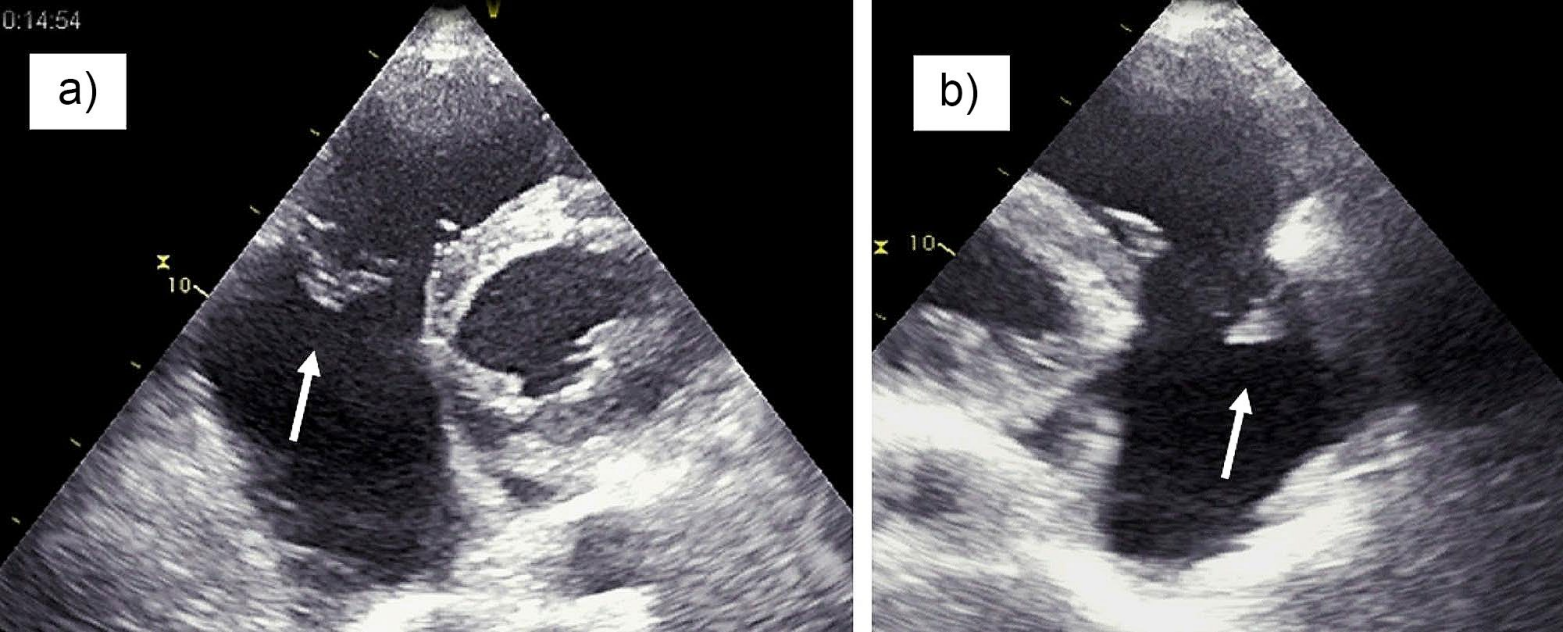
Transthoracic echocardiography showing flail anterior tricuspid leaflet (arrows): (**a**) parasternal short axis view and (**b**) modified parasternal long axis view

**Fig. 3:**
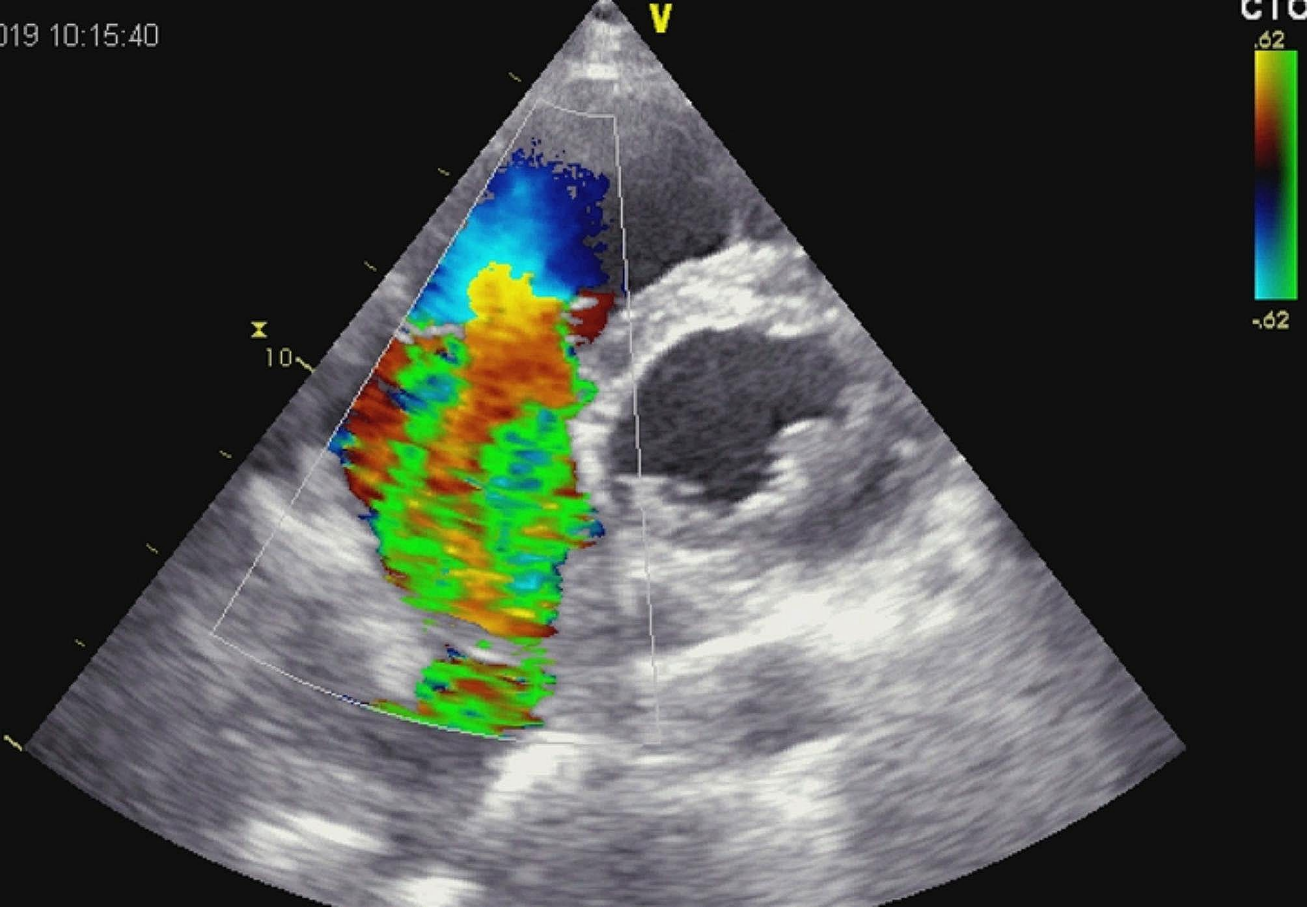
Massive tricuspid regurgitation, shown by transthoracic colour doppler (parasternal short axis view)

**Fig. 4:**
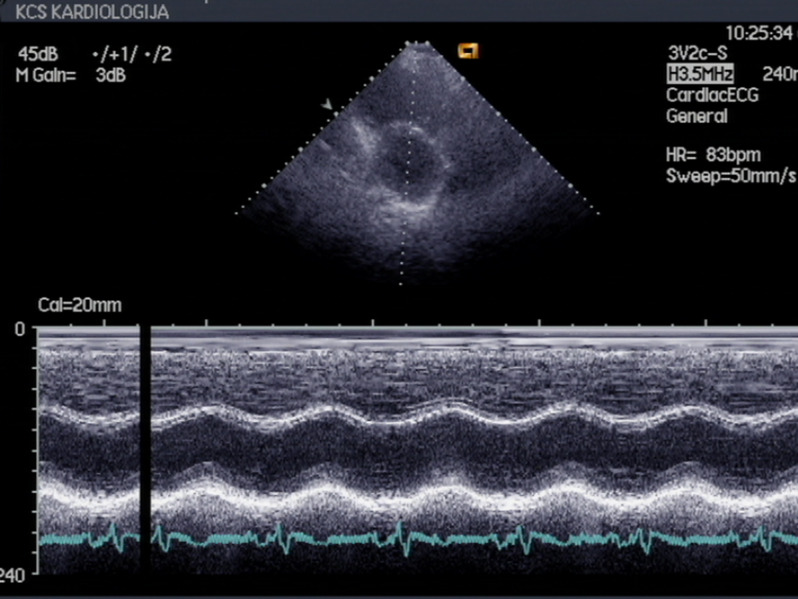
Transthoracic echocardiography shows left ventricle in parasternal short axis view with paradoxical movements of the septum

A 58-year-old patient was admitted to the hospital due to chest pain, dyspnea and fatigue on physical exertion. The symptoms started two weeks before admission. His previous history was unremarkable, except for a car accident 10 years ago, with chest trauma and fracture of two ribs. On physical examination, the patient was cyanotic, with signs of right ventricular failure and pansystolic murmur at the lower left sternal border; the blood pressure was 110/80 mmHg, heart rate was 110/min. The electrocardiogram (ECG) showed a right bundle branch block (Fig. 1). TTE revealed flail of the anterior leaflet of tricuspid valve due to chordal rupture, leaving the anterior half of the leaflet completely unsupported. Also, there was a moderate tethering of the septal and posterior leaflet. (Fig. 2, Supplementary material 1 and 2). Massive tricuspid regurgitation, registered by Colour Doppler (Fig. 3, Supplementary material 3), was considered to be post-traumatic, based on the exclusion of other causes. Ebstein anomaly was excluded by echocardiography (the septal displacement index of the insertion of tricuspid to mitral leaflet was 0.76 cm/m2 (i.e. < 0.8 cm/m2). Other causes were ruled out by normal values of laboratory blood tests, including inflammatory markers and blood cultures (infective endocarditis, carcinoid), as well as the absence of any gastrointestinal symptoms, invasive cardiac intervention or radiation therapy. Detailed TTE and transoesophageal echocardiography (TEE) examination also revealed enlarged right ventricle (RV diastolic diameter was 5,1 cm from the PLAX view, compared to LV diameter of 4,8 cm), moderate reduction of right ventricular function, and TV annular dilatation (4,4 cm) with reduced motion amplitude. Left ventricular shape and dimensions were within normal limits and no wall motion abnormalities were observed, apart from paradoxical movements of the septum, caused by right ventricular overload. There was no significant pericardial effusion. (Fig. 4, Supplementary material 4).

**Fig. 5:**
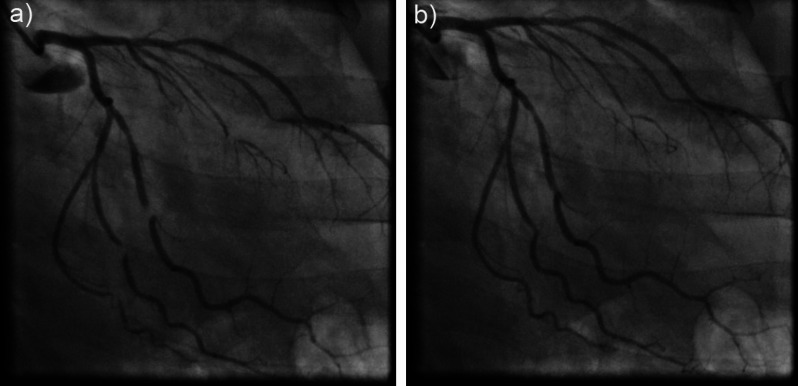
Coronary angiography showing multiple dynamic stenoses of left coronary arteries, caused by the thick edge of the ruptured pericardium: (**a**) obstructed flow; (**b**) resolved flow

**Fig. 6:**
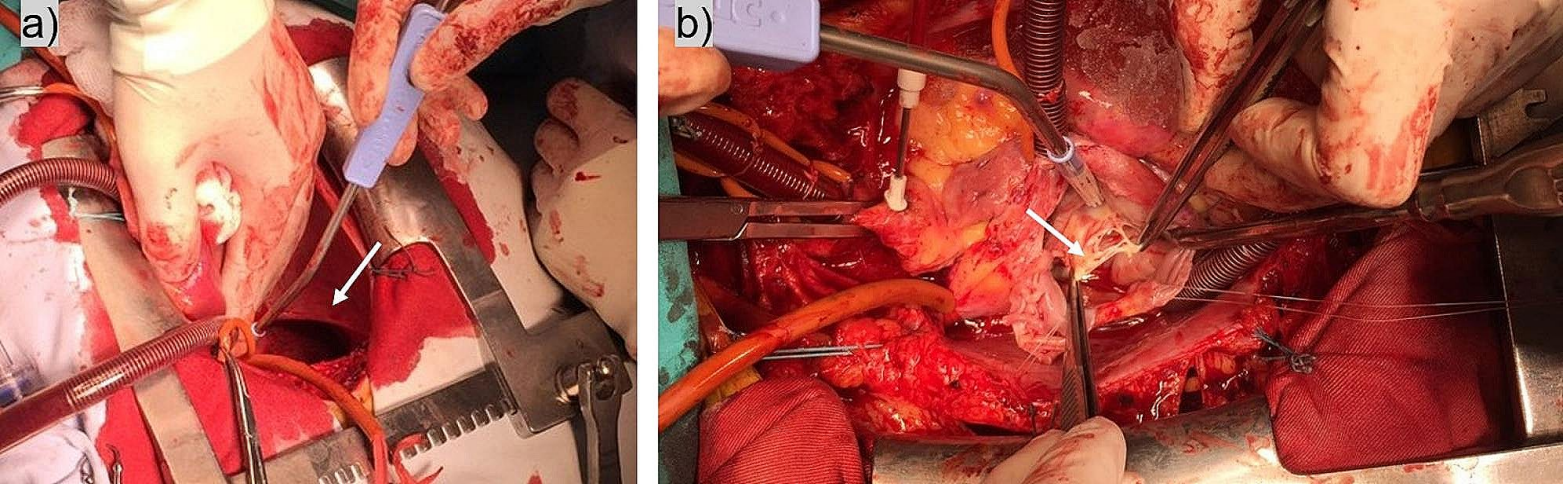
Intraoperative finding: (**a**) pericardial rupture (arrow); (**b**) anterior leaflet of the tricuspid valve with the rupture of the main common chord (arrow)

After 10 days of intensive heart failure therapy, the patient was transferred to the cardiac surgery department for reconstruction of the tricuspid valve. Coronary angiography, performed prior to cardiac surgery, revealed the presence of an unusual finding of multiple dynamic stenotic lesions at the same level of all left-sided coronary vessels, predominantly on the first, second and third obtuse marginal branches of the circumflex coronary artery (Fig. 5, Supplementary material 5). After medial sternotomy, an old pericardial rupture with thick edge was observed (Fig. 6a). Pericardial opening was measuring 7 × 6 cm in size and the heart was herniated through the pericardial defect. The edge of the ruptured pericardium compressed the coronary arteries along the line, which was in accordance with the previous coronary angiographic findings. The heart was returned to normal position and the pericardial rupture was sutured. Intraoperatively, it was confirmed that the anterior leaflet of the tricuspid valve completely lost its support, due to the rupture of the main common chord at the level of papillary muscle (Fig. 6b). Based on the measures calculated by transesophageal echocardiography, the CorMatrix patch was constructed and a tube was formed for the reconstruction of the tricuspid valve. The leaflets were then excised and the CorMatrix patch was sutured in three places to the bases of the papillary muscles and proximally to the tricuspid annulus. The specific tricuspid surgery with Cor Matrix patch was performed because the leaflet tissue was insufficient in size to cover the valve area and to perform neochordal implantation.

**Fig. 7:**
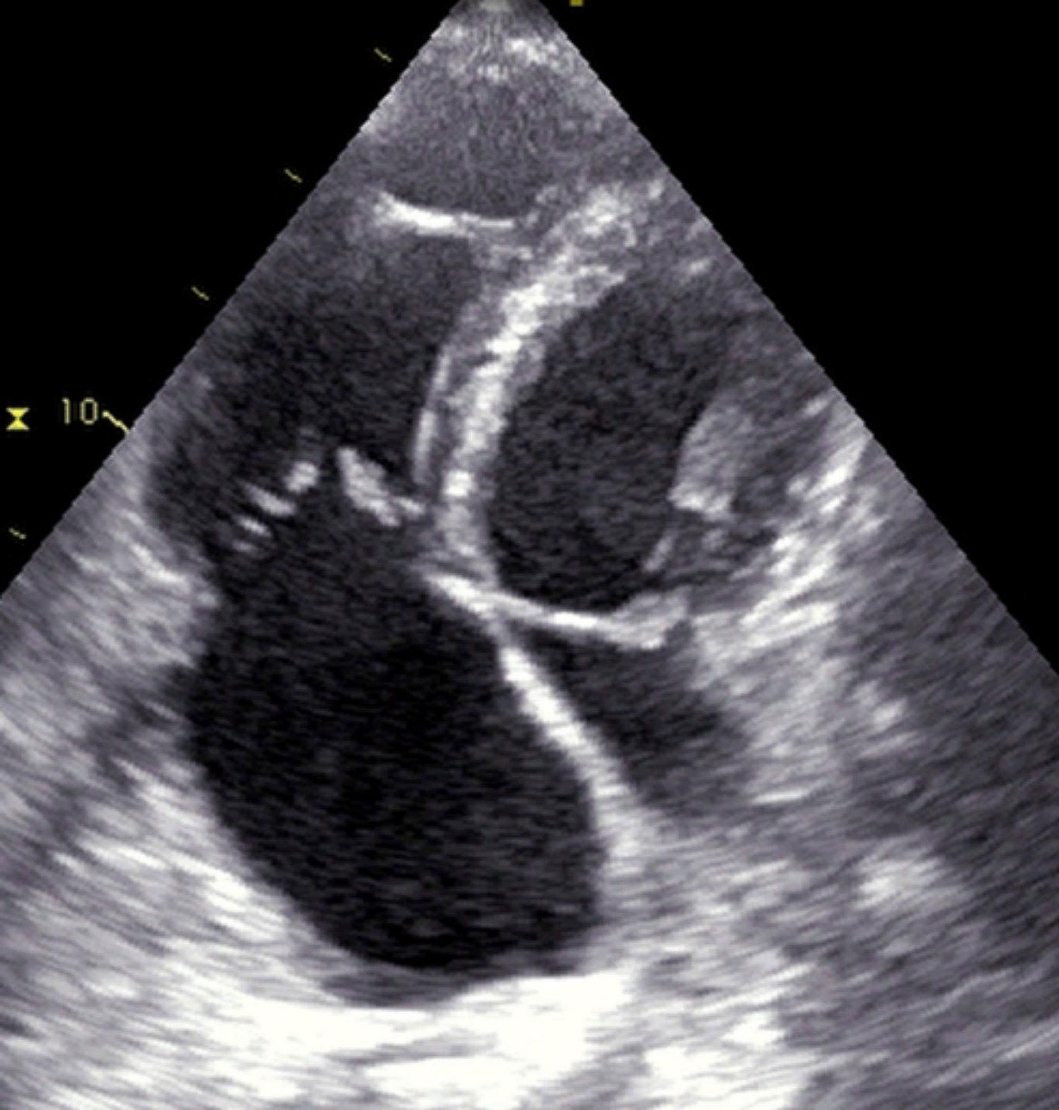
Postoperative TTE showing reconstructed tricuspid valve (modified apical four chamber view)

On control transthoracic echocardiography, a tube connected to the annulus and the base of the papillary muscles was confirmed (Fig. 7, Supplementary material 6). Mild residual tricuspid regurgitation persisted, with right ventricular systolic pressure 38 mmHg. After recovery, the patient was discharged home, without symptoms and signs of right heart failure.

## Discussion and conclusions

Although chest injuries in a car accident are common, injuries of valvular structures are very rare (less than 1%) and usually present late [1–3]. The right ventricle is located just behind the sternum and therefore prone to injury, caused by the pressure forces to the front or back of the chest. The mechanism of tricuspid valve injury is usually due to a deceleration force transmitted to the chest and heart, especially if the force acted during late diastole, thus leading to a rapid increase in right ventricular intracavitary pressure, which may lead to the rupture of the papillary muscle or tendinous chords [1, 4]. The mechanism of delayed rupture of the tricuspid valve is usually due to contusion of the papillary muscle, followed by haemorrhage, inflammation, and necrosis that can lead over time to rupture of the valvular apparatus [5, 6]. Rupture of the papillary muscle usually presents acutely and is therefore treated very quickly surgically [7]. In contrast, rupture of tendinous chords has a much milder clinical course and often remains unrecognized after the injury [8]. Therefore, wide time periods are described in the literature during which the rupture of tricuspid valve was detected and corrected [7–10]. Our patient belongs to the group of late ruptures, with significant tricuspid regurgitation and signs of right ventricular failure. We may assume that the rupture of tricuspid chords occurred earlier (before the patient reported symptoms), so that tricuspid regurgitation (and volume overload) lasted longer and caused both - degeneration of the anterior leaflet and RV dilatation and dysfunction, leading to RV-related heart failure. When these symptoms became prominent, together with symptoms caused by the compression of the edges of the ruptured pericardium on the coronary arteries, the patient presented to the emergency department. As the anterior leaflet of tricuspid valve suffered significant degeneration due to loss of support and huge motions in large blood stream, it became shortened and thickened.

Myocardial injuries, in addition to rupture of the valvular apparatus, may include myocardial contusion, rupture of a free wall or septum, and pericardial effusion. The highest percentage of traumatic injuries to the valvular apparatus was observed on the aortic and mitral valves, due to higher pressures in the left heart [11].

Echocardiography has a significant role, especially in patients with minimal clinical symptoms. This technique also serves to adequately describe anatomical disorders that occur after an injury, which is of great importance to the cardiac surgeon, in order to select an adequate surgical technique. A limitation of TTE is the fact that these patients usually have significant chest injuries, including haemothorax and pneumothorax, which makes their echocardiographic windows less adequate for interpretation compared to patients without chest injury. Prolonged haemodynamic instability of the patient prompts the physician to repeat the TTE examination or consider a TEE [7]. Both TTE and TEE may not be ideal in some cases [9]. In our patient, pericardial rupture with LV protrusion was not seen on echocardiography (probably because of the elastic forces of the LV wall), which indicates the necessity of other diagnostic procedures, such as chest computed tomography (CT) scan and cardiac magnetic resonance (CMR) imaging in symptomatic post-trauma patients, which is advised by other authors, as well [12].

The rupture of the pericardium in blunt chest trauma is also very rare [13]. Deceleration forces are usually responsible for the occurrence of pericardial defect, since the base of the heart is more fixed to the pulmonary vasculature and aorta, while the apex is more mobile, causing the rupture mostly on the lateral side of pericardium [13]. Pericardial rupture is seen in less than 0.5% of patients presenting after blunt trauma, and cardiac herniation through a pericardial defect is a potential complication of this injury [14]. In some occasions, herniation of the heart can be asymptomatic and go unrecognized [15]. On the other hand, major cardiac herniation can cause torsion of the great vessels, included inferior vena cava and strangulation of the herniated heart, causing cardiogenic shock and sudden death [14, 16, 17]. Also, if pneumopericardium occurs, air within a limited potential space can result in cardiac tamponade and hemodynamic instability [17].

Pericardial rupture is difficult to diagnose by echocardiographic techniques because of tiny structure of pericardium. Some indirect signs such as pneumopericardium or hemopericardium might be of help but could not prove definite diagnosis [16]. Chest CT scan enables timely recognition of pericardial rupture. The defect in the pericardium outlined by air may be directly visible on CT. If there is accompanying cardiac herniation, constriction by the pleuro-pericardial defect can be visible like a collar or waist [15, 17]. Also, cardiac tamponade can be seen, as compression of the heart chambers by the air in the pericardial space which results in a small heart size [18].

CMR imaging plays an important role in the assessment of pericardial injuries and cardiac herniation. The best way to visualize the pericardium is by using T1 weighted imaging during systole [19, 20]. This visualization method could make very good distinction between the pericardial and myocardial tissue. Besides that, CMR imaging is superior to CT because it generates motion pictures and can estimate regional wall motion abnormalities. These cine MR images could identify motions of the heart which is dislocated from the pericardial sac through the pericardial tear, indicating possible dynamic obstruction of the ventricles, as well as major blood vessels. However, even the CMR imaging has limitations. The parietal pericardium may be incompletely visualized, especially over left sided chambers, where pericardial rupture happens very often, because of scarcity of surrounding fat [21]. The cardiac herniation visualised by the CMR imaging is often intermittent and limited by the changes in the decubital position of the patient [21].

The question of when to operate the patient with traumatic tricuspid regurgitation that occurred during a chest injury remains open. The best results were achieved with early use of surgical techniques in patients with severe tricuspid valve regurgitation [22]. In contrast, in patients who were presented to a cardiac surgeon late, atrophy of the papillary muscle and chords and significantly increased amplitude of tricuspid valve leaflet movement were noted. Therefore, it is considered that surgical treatment of these patients, before the development of right ventricular failure, prevents further complications and maintains a stable sinus rhythm. Reparative techniques for treating traumatic tricuspid valve injuries today involve the use of synthetic materials to replace the ruptured chord or papillary muscle [22–24].

Traumatic injuries of the tricuspid valve and pericardium are often unrecognized in a timely manner, leading to late complications and right heart failure. Transthoracic and transoesophageal echocardiography play a crucial role in the recognition and proper treatment of these entities, though cardiac CMR may be needed in some cases. Early surgical treatment of unstable patients with severe tricuspid regurgitation prevents further complications and maintains a stable sinus rhythm.

### Electronic supplementary material

Below is the link to the electronic supplementary material.

**Supplementary Material 1:** Transthoracic echocardiography showing flail of the anterior leaflet of tricuspid valve (parasternal short axis view)

**Supplementary Material 2:** Transthoracic echocardiography showing flail of the anterior leaflet of tricuspid valve (modified parasternal long axis view)

**Supplementary Material 3:** Massive tricuspid regurgitation, shown by Transthoracic Colour Doppler (parasternal short axis view)

**Supplementary Material 4:** Transthoracic echocardiography showing left ventricle in short axis view

**Supplementary Material 5:** Coronary angiography showing dynamic stenoses of left coronary arteries caused by the thick edge of the ruptured pericardium with obstructed and resolved flow

**Supplementary Material 6:** Postoperative transthoracic echo showing reconstructed tricuspid valve (modified apical four chamber view)

## Data Availability

Please contact the corresponding author regarding data availability.

## Abbreviations

CMR: Cardiac magnetic resonance
CT: computed tomography
ECG: Electrocardiogram
LV: left ventricle
PLAX view: parasternal long axis view
RV: right ventricle
TEE: Transoesophageal echocardiographic examination
TEE: Transthoracic echocardiography
TV: tricuspid valve

## References

[1] Lin S-J, Chen C-W, Chou C-J, Liu K-T, Su H-M, Lin T-H (2006). Traumatic tricuspid insufficiency with Chordae Tendinae rupture: a Case Report and Literature Review. Kaohsiung J Med Sci.

[2] Veld MAH, Craft CA, Hood RE (2018). Blunt Cardiac Trauma Review Cardiol Clin.

[3] Saar S, Lomp A, Laos J, Mihnovitš V, Šalkauskas R, Lustenberger T (2017). Population-based autopsy study of traumatic fatalities. World J Surg.

[4] Thekkudan J, Luckraz H, Ng A, Norell M (2012). Tricuspid valve chordal rupture due to airbag injury and review of pathophysiological mechanisms. Interact Cardiovasc Thorac Surg.

[5] Kulik A, Al-Saigh M, Yelle J-D, Rubens FD (2006). Subacute Tricuspid Valve Rupture after traumatic cardiac and pulmonary contusions. Ann Thorac Surg.

[6] Jin H-Y, Jang J-S, Seo J-S, Yang T-H, Kim D-K, Kim D-K (2011). A case of traumatic tricuspid regurgitation caused by multiple papillary muscle rupture. J Cardiovasc Ultrasound.

[7] Jung H, Cho JY, Kim G-J, Lee Yok, Lim KH, Hong SW (2019). Traumatic severe tricuspid regurgitation diagnosis after the progression of right ventricle function deterioration. Trauma Case Rep.

[8] Cheng Y, Yao L, Wu S (2017). Traumatic tricuspid regurgitation. Int Heart J.

[9] Ismailov RM, Weiss HB, Ness RB, Lawrence BA, Miller TR (2005). Blunt cardiac injury associated with cardiac valve insufficiency: trauma links to chronic disease?. Injury.

[10] Hill GED, Thorsen TN, Goelz AP, Miller RE, Almassi GH, Pagel PS (2019). A rare consequence of Remote Blunt chest trauma. J Cardiothorac Vasc Anesth.

[11] Varahan SL, Farah GM, Caldeira CC, Hoit BD, Askari AT (2006). The double jeopardy of blunt chest trauma: a case report and review. Echocardiogr Mt Kisco N.

[12] Avegliano G, Corneli M, Conde D, Ronderos R (2014). Traumatic rupture of the tricuspid valve and multi-modality imaging. Cardiovasc Diagn Ther.

[13] Gao R, Jia D, Zhao H, WeiWei Z, Yangming WF (2018). A diaphragmatic hernia and Pericardial Rupture caused by Blunt Injury of the chest: a Case Review. J Trauma Nurs off J Soc Trauma Nurses.

[14] Chughtai T, Chiavaras MM, Sharkey P, Shulman H, Miller HA (2008). Pericardial rupture with cardiac herniation. Can J Surg.

[15] Watkins BM, Buckley DC, Peschiera JL (1995). Delayed presentation of pericardial rupture with luxation of the heart following blunt trauma: a case report. J Trauma.

[16] Guenther T, Rinderknecht T, Phan H, Wozniak C, Rodriquez V (2020). Pericardial rupture leading to cardiac herniation after blunt trauma. Trauma Case Rep.

[17] Nassiri N, Yu A, Statkus N, Gosselin M (2009). Imaging of cardiac herniation in traumatic pericardial rupture. J Thorac Imaging.

[18] Verma N, Robinson JD, Gunn ML (2018). Pericardial rupture and cardiac herniation in blunt trauma. Radiol Case Rep.

[19] Adams A, Fotiadis N, Chin JY, Sapsford W, Brohi K (2012). A pictorial review of traumatic pericardial injuries. Insights Imaging.

[20] Scagliola R, Seitun S, Rosa GM (2021). Myocardial crypts, recesses, and outpouchings: it is time to clarify. Pol Arch Intern Med.

[21] Bogaert J, Francone M (2013). Pericardial disease: value of CT and MR imaging. Radiology.

[22] Ma W-G, Luo G-H, Sun H-S, Xu J-P, Hu S-S, Zhu X-D (2010). Surgical treatment of traumatic tricuspid insufficiency: experience in 13 cases. Ann Thorac Surg.

[23] Zhang Z, Yin K, Dong L, Sun Y, Guo C, Lin Y (2017). Surgical management of traumatic tricuspid insufficiency. J Card Surg.

[24] Fender EA, Zack CJ, Nishimura RA (2018). Isolated tricuspid regurgitation: outcomes and therapeutic interventions. Heart Br Card Soc.

